# Evaluation of Sofosbuvir (β-D-2′-deoxy-2′-α-fluoro-2′-β-C-methyluridine) as an inhibitor of Dengue virus replication^**#**^

**DOI:** 10.1038/s41598-017-06612-2

**Published:** 2017-07-24

**Authors:** Hong-Tao Xu, Susan P. Colby-Germinario, Said A. Hassounah, Clare Fogarty, Nathan Osman, Navaneethan Palanisamy, Yingshan Han, Maureen Oliveira, Yudong Quan, Mark A. Wainberg

**Affiliations:** 1 McGill University AIDS Centre, Lady Davis Institute for Medical Research, Jewish General Hospital, Montreal, Quebec, Canada; 2 Department of Medicine, McGill University, Montreal, Quebec, Canada; 3 Department of Microbiology and Immunology, McGill University, Montreal, Quebec Canada; 4 HBIGS, University of Heidelberg, Heidelberg, Germany

## Abstract

We evaluated Sofosbuvir (SOF), the anti-hepatitis C virus prodrug of β-d-2′-deoxy-2′-α-fluoro-2′-β-C-methyluridine-5′-monophosphate, for potential inhibitory activity against DENV replication. Both cell-based and biochemical assays, based on use of purified DENV full-length NS5 enzyme, were studied. Cytopathic effect protection and virus yield reduction assays confirmed that SOF possessed anti-DENV activity in cell culture with a 50% effective concentration (EC_50_) of 4.9 µM and 1.4 µM respectively. Real-time RT-PCR verified that SOF inhibits generation of viral RNA with an EC_50_ of 9.9 µM. Purified DENV NS5 incorporated the active triphosphate form (SOF-TP) into nascent RNA, causing chain-termination. Relative to the natural UTP, the incorporation efficiency of SOF-TP was low (discrimination value = 327.5). In a primer extension assay, SOF-TP was active against DENV NS5 wild-type polymerase activity with an IC_50_ of 14.7 ± 2.5 µM. The S600T substitution in the B Motif of DENV polymerase conferred 4.3-fold resistance to SOF-TP; this was due to decreased incorporation efficiency rather than enhanced excision of the incorporated SOF nucleotide. SOF has antiviral activity against DENV replication. The high discrimination value in favor of UTP in enzyme assays may not necessarily preclude antiviral activity in cells. SOF may be worthy of evaluation against severe DENV infections in humans.

## Introduction

Dengue fever is caused by the four serotypes of Dengue virus (DENV) and is the most prevalent arthropod-borne viral disease of humans with major impact on public health^1^. The global incidence of DENV infection has grown dramatically in recent years and about half of the world’s population is now at risk^2^. Infection with any of the DENV serotypes may result in a wide spectrum of clinical symptoms ranging from a mild flu-like syndrome to the most severe forms of Dengue haemorrhagic fever (DHF), that are characterized by coagulopathy, increased vascular fragility, and permeability. DHF may progress to hypovolemic shock and dengue shock syndrome (DSS)^1, 3^. DENV causes half a million cases of DHF/DSS and 22,000 deaths annually and no specific anti-DENV therapeutic is currently available^2^.

DENV is classified within the family Flaviviridae in the genus Flavivirus^4^. This genus also contains several other major human pathogens such as Japanese Encephalitis virus (JEV), West Nile virus (WNV), Yellow Fever virus (YFV) Tick-Borne Encephalitis virus (TBEV) and Zika virus (ZIKV)^4^. Flaviviruses share similar genome structures and replication strategies and their viral genomes involve a ~11 kb plus-sense single-stranded RNA with a type-1 cap structure that is followed by a strictly conserved dinucleotide sequence “AG”, i.e., 5′-m7GpppAm2′-O-G-3′^5^. Flavivirus genomes encode a large polyprotein precursor that is co- and post-translationally processed by viral and cellular proteases into three structural (capsid, C; membrane precursor, prM; and envelope, E) and seven non-structural (NS) proteins (NS1, NS2A, NS2B, NS3, NS4A, NS4B and NS5). The NS proteins co-translationally assemble on endoplasmic reticulum membranes to form a competent replication complex (RC). The NS3 and NS5 proteins are central to viral replication since these proteins are associated with most of the catalytic activities required to both cap and replicate viral RNA. In similar fashion to the NS5B of Hepatitis C virus (HCV) the flavivirus NS5 initiates RNA synthesis by a *de novo* (primer-independent) mechanism^6, 7^. The RNA-dependent RNA polymerase (RdRp) activity of NS5 is crucial for viral replication. The absence of a mammalian host cellular enzyme equivalent may mean that inhibitors of NS5 RdRp activity may not encounter problems of toxicity^8, 9^.

The DENV NS5 comprises an N-terminal methyl-transferase domain (NS5MTase) and a C-terminal RdRp domain (NS5POL). The NS5 is the largest (~900 amino acids in size) and most conserved NS protein with a sequence identity of around 70% among the four DENV serotypes. The crystal structures of NS5 and the NS5POL domain have been solved and display a classic right hand global structure with fingers, palm and thumb domains^10–12^. A high degree of structural conservation within the NS5POL domain was observed among Flaviviridae family members^9^. Among these domains, the 6 conserved motifs A-F which play key roles in RNA, rNTP, and metal-ion binding and catalysis have been well characterized in DENV RdRp^11, 12^. The amino acids involved at the catalytic site of DENV RdRp are located within motif A (aspartate at position 533, D533) and the catalytic triad GDD at positions 662–664 in motif C. These aspartate residues are involved in the coordination of two divalent metal ions that are essential for nucleotidyl transfer, termed the “two-metal-ion mechanism”^13^.

Two major classes of DENV RdRp inhibitors, nucleoside/nucleotide analogue inhibitors (NIs) and non-nucleoside analogue inhibitors (NNIs), have been investigated (for reviews see refs 8, 9, 14, 15). NIs require intracellular phosphorylation to the active 5′-triphosphate form by cellular kinases, then bind at the enzyme active site and compete with the natural substrate for incorporation, and this is followed by chain termination^16, 17^. NNIs do not require intracellular activation and provide an alternative inhibitory mechanism by binding to allosteric sites away from the active site of the RdRp to interfere with RNA synthesis^8, 9, 15, 18^. An adenosine analog, NITD-008, was shown to possess antiviral activity in a mouse model of lethal DENV infection and prevented infected mice from death. However, further development of this compound was stopped because of a narrow therapeutic window and toxicity^9, 15, 17^. Until now, no NIs or NNIs have been approved for clinical treatment of DENV infections. However, drugs in the class of both NI and NNI have been approved for clinical treatment of HCV infection, for review see refs 19, 20.

Sofosbuvir, the phosphoramidate prodrug of β-D-2′-deoxy-2′-α-fluoro-2′-β-C-methyluridine-5′-monophosphate (2′-F-2′-C-Me-UMP, SOF) acts after metabolism to the active triphosphate form β-D-2′-deoxy-2′-α-fluoro-2′-β-C-methyluridine-5′-triphosphate (2′-F-2′-C-Me-UTP, SOF-TP) as a non-obligate chain terminator of HCV NS5B polymerase activity^21–24^. SOF has been approved by the Food and Drug Administration (FDA) for treatment of hepatitis C virus infections. This same class of 2′-modified nucleotide analogs has also been demonstrated to inhibit the viral RNA polymerases of other positive-stranded RNA viruses, (for review see ref. 25), including Zika virus, which is closely related to DENV, both in cell culture and in an animal model^26, 27^. Direct inhibition of Zika virus RNA polymerase has been demonstrated in *in vitro* RNA polymerase assays using purified RC from infected cells and recombinant RdRp purified from bacteria^27, 28^. However, the anti-DENV activity of SOF in cell culture has not been reported. Here, we show that SOF possesses anti-DENV activity in cell culture and also provide a detailed biochemical analysis of the active triphosphate SOF-TP as an inhibitor of DENV NS5 polymerase activity was not performed.

## Results

### Sofosbuvir exhibits potent anti-Dengue virus activity in cell-based assays

**Figure 1:**
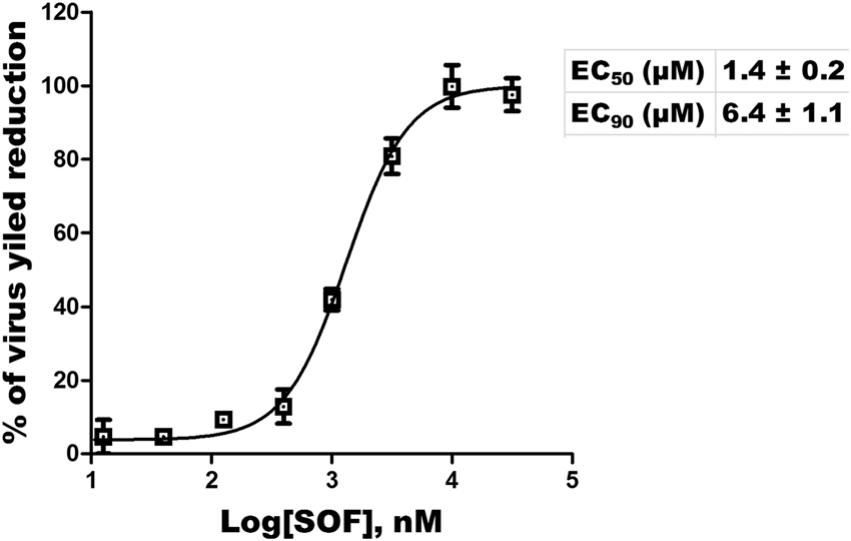
SOF is an inhibitor of DENV replication as determined by virus yield reduction assay. Huh7 cells infected by DENV were treated with increasing concentrations of SOF. Supernatants were collected 48 h post infection and virus titers were determined in Huh7 cells by TCID_50_ analysis. The percentage of virus yield reduction was calculated with respect to no-compound control experiments. Data points represent an average of three experiments, and error bars represent standard deviations.

To evaluate the anti-DENV activity and relative toxicities of SOF in cell culture, we used the well-established cytopathic effect (CPE) protection assay using a Promega Viral ToxGlo Assay kit^27, 29^ and Huh7 cells. DENV-infected cells were treated with increasing concentrations of SOF or control compound and protection from CPE was measured. The broad-spectrum antiviral agent mycophenolic acid (MPA)^30, 31^ was used as a positive control of inhibition of DENV replication. The data (Table 1) show that SOF was able to inhibit DENV infectivity at an EC_50_ of 4.9 µM, whereas the EC_50_ of the control compound MPA was 0.7 µM, in agreement with previous reports^29, 31^. The CC_50s_ of SOF and MPA were both >100 µM as assayed in Huh7 cells. Thus, the selectivity index (SI) of SOF was over 20, which indicates that SOF is a potent anti-DENV compound in our cell culture assay. To confirm this observation, an *in vitro* DENV yield reduction assay was performed. The EC_50_ of SOF was found to be 1.4 ± 0.2 µM and the EC_90_ value was 6.4 ± 1.1 µM (Fig. 1), which indicates that the anti-DENV potency of SOF is even higher than observed in the CPE protection assay.

**Table 1:** Antiviral activity of test compounds against Dengue virus infection in cell culture.

	EC_50_ (µM)^a^	CC_50_ (µM)^b^	SI^c^
SOF	4.9 ± 1.3	>100	>20
MPA	0.7 ± 0.4	>100	>100

^a^The 50% effective concentration (EC_50_) was determined in Huh7 cells using the Promega Viral ToxGlo Assay. Data represent the means ± standard deviation (SD) of 3 independent experiments. ^b^The 50% cytotoxic concentration (CC_50_) was determined in Huh7 cells using the Viral ToxGlo Assay. ^c^SI: selectivity index, as determined by the ratio of CC_50_ to EC_50_.

**Figure 2:**
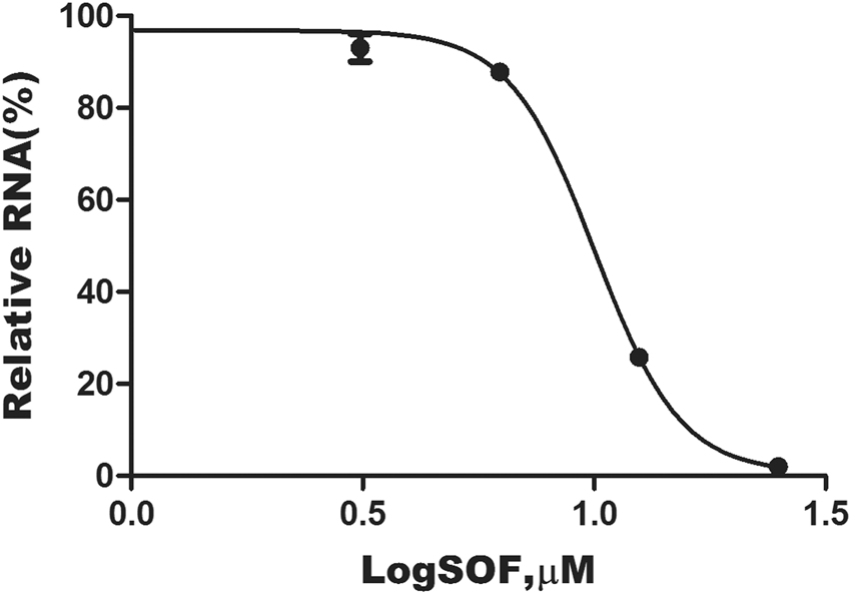
Dose-response curve showing inhibition of DENV RNA production by SOF. The inhibitory effect of SOF on viral RNA levels was determined by qRT-PCR assay. Relative levels of viral RNA were calculated as a percentage of levels in untreated control cells. The data shown are from experiments run in duplicate. The mean of EC_50_ values and standard deviation (SD) determined from the results of three independent experiments was 9.9 ± 1.2 µM.

To further verify the anti-DENV activity of SOF in cell culture, we examined the inhibition of DENV RNA production by SOF using qRT-PCR assay. In this experiment, we observed a significant reduction in DENV RNA production by SOF in a dose-dependent manner relative to untreated control cells. The EC_50_ of SOF was 9.9 ± 1.2 µM (Fig. 2), about double the EC_50_ obtained using the Viral ToxGlo Assay. Similar differences were observed in a recent report showing that SOF is more potent at inhibiting Zika virus-induced cell death and viral infectivity than viral RNA production^28^. These data suggest that a fraction of virus particles containing genomic RNA may still be produced following SOF exposure but may not be able to efficiently infect new cells and induce cytopathicity.

### Purification of recombinant DENV2 full-length NS5

The DENV NS5MTase domain has been observed to have positive effects on NS5 polymerase activity and improve enzyme steady-state constants^11, 16, 32–34^. Thus, we cloned the DENV2 full-length NS5 coding sequence into the bacterial expression vector pET21a using a C-terminal his6-tag. Recombinant full-length NS5 wild-type and mutant enzyme containing a S600T substitution were purified to >95% homogeneity under native conditions by immobilized metal ion affinity and size-exclusion chromatography as demonstrated by SDS-PAGE (data not shown).

### SOF-TP can be incorporated into nascent RNA and cause chain termination by DENV NS5

To verify that SOF-TP can be incorporated into a nascent RNA chain by DENV NS5 and cause chain termination, we performed primer extension experiments using purified recombinant DENV NS5 enzyme, RNA duplex P10/T14 and SOF-TP in the presence or absence of the next incoming nucleotide, i.e., CTP. Chain termination refers to the incapacity of a polymerase, following incorporation of a nucleotide analog, to continue to synthesize nucleic acid in the presence of the next correct nucleotide. Classical obligate chain terminators lack a 3′-hydroxyl group and differ from the 2′- modified nucleotide analogues such as SOF that retain a functional 3′-hydroxyl. It has therefore been proposed that the latter class of nucleotide analogues causes chain termination by steric hindrance exerted through 2′-C-methyl or 2′-fluoro groups (for reviews, see refs 19, 20).

**Figure 3:**
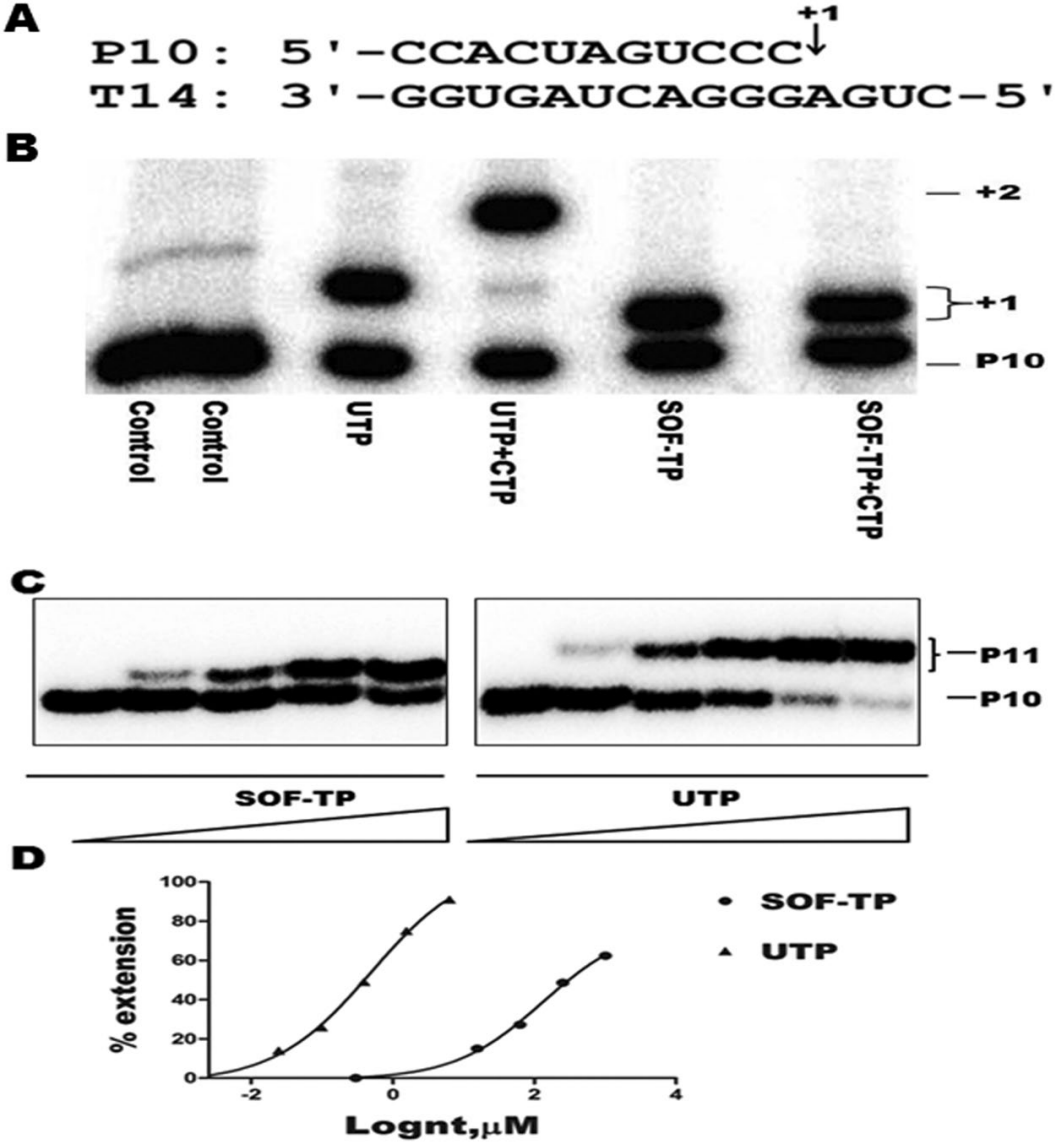
Comparison of incorporation efficiency of UTP and SOF-TP by DENV NS5. (**A**) Sequences of the RNA primer P10 and the RNA template T14 used in the primer extension assay. +1 indicates the position of the first nucleotide to be incorporated. (**B**) Parallel comparisons of incorporation of natural UTP (5 µM for 10 min) or nucleotide analogue SOF-TP (200 µM for 50 min) into RNA and chain-termination by purified recombinant DENV NS5 were performed at 30 °C in a primer extension assay. +1 and +2 indicate the positions of the first and the next nucleotides to be incorporated. P10 indicates the position of the 5′-end radiolabeled P10 primer. Control represents reaction without addition of nucleotide or nucleotide analogue as a negative control. Of note, the migration of extended primer after incorporation of SOF-TP was faster than that seen after incorporation of the natural substrate UTP. (**C**) Single nucleotide incorporation reactions were performed at 30 °C in a standard primer extension assay using RNA duplex P10/P11 and DENV NS5 in the presence of variable concentrations of natural nucleotide UTP or the nucleotide analogue SOF-TP. Reaction products were analyzed using denaturing polyacrylamide gel electrophoresis (20% acylamide, 7 M Urea) and by phosphorimaging. The positions of P10 and extended P11 are indicated on the right of the panels. (**D**) Quantitative analysis of UTP and SOF-TP incorporation in panel A. The incorporation efficiency was evaluated based on the extension of 5′-end radiolabeled P10 to P11. The measured K_1/2_ values, i.e., concentrations of nucleotides (nt) at which half of the total percentage of the P11 product was formed for UTP and SOF-TP were 0.4 μM and 131 μM, respectively.

In our primer extension assay, we have shown that DENV NS5 extended the P10 primer by one nucleotide (+1) (Fig. 3A) in the presence of either the natural UTP or SOF-TP in the absence of the next correct nucleotide, CTP (Fig. 3B). Of note, the extended primer following incorporation of SOF-TP migrated much faster than did that extended by UTP. In the presence of the next correct nucleotide CTP, the P10 primer was further extended to the +2 position in the case of the natural UTP. In comparison, the incorporation of SOF-TP fully prevented the extension of the primer to the +2 position (Fig. 3B). These data confirm that SOF-TP can be recognized by DENV NS5 as an effective nucleotide substrate, that is incorporated into the nascent RNA chain to cause the termination of RNA synthesis. This is in agreement with recent reports that showed that DENV polymerase is able to use SOF-TP as a nucleotide substrate in primer extension assays^35, 36^. Our molecular modelling data below further support the notion that SOF-TP can be accommodated into the active site of DENV polymerase.

### DENV NS5 incorporates SOF-TP with lower efficiency than the natural substrate UTP in a single-nucleotide incorporation assay

The relative incorporation efficiency of nucleotide analogs versus natural ribonucleotides may be relevant to the ability of certain analogs to inhibit RdRp activity^22, 36^. In this study, the relative incorporation efficiencies of SOF-TP and the natural substrate UTP were compared in single nucleotide incorporation reactions. These reactions were performed at 30 °C as standard primer extension assays by incubation of DENV NS5 and the P10/P11 RNA duplex in the presence of variable concentrations of the natural nucleotide UTP or SOF-TP. Reaction products were analyzed using denaturing polyacrylamide gel electrophoresis and phosphorimaging. K_1/2_ values, i.e., concentrations of nucleotides (nt) at which half of the total percentage of the P11 product for UTP or SOF-TP were formed, were determined by plotting the data against a tested nucleotide concentration and analyzed by non-linear regression using GraphPad Prism5.0 software (GraphPad Software, San Diego, CA). The gels for SOF-TP or UTP incorporation shown in Fig. 3C were used for the band intensity quantification. In this assay the measured K_1/2_ values of UTP and SOF-TP were 0.4 μM and 131 μM, respectively (Fig. 3D). Thus, the discrimination value which was calculated by dividing the K_1/2_ of SOF-TP by the K_1/2_ of UTP is 327.5. This result suggests that SOF-TP is a less efficient nucleotide substrate for DENV NS5 than UTP in single nucleotide incorporation assays. A recent report also showed a high discrimination value for SOF-TP in primer extension assays with both ZIKV and DENV NS5 enzymes^36^.

### Inhibitory effect of SOF-TP on DENV RdRp activity

**Figure 4:**
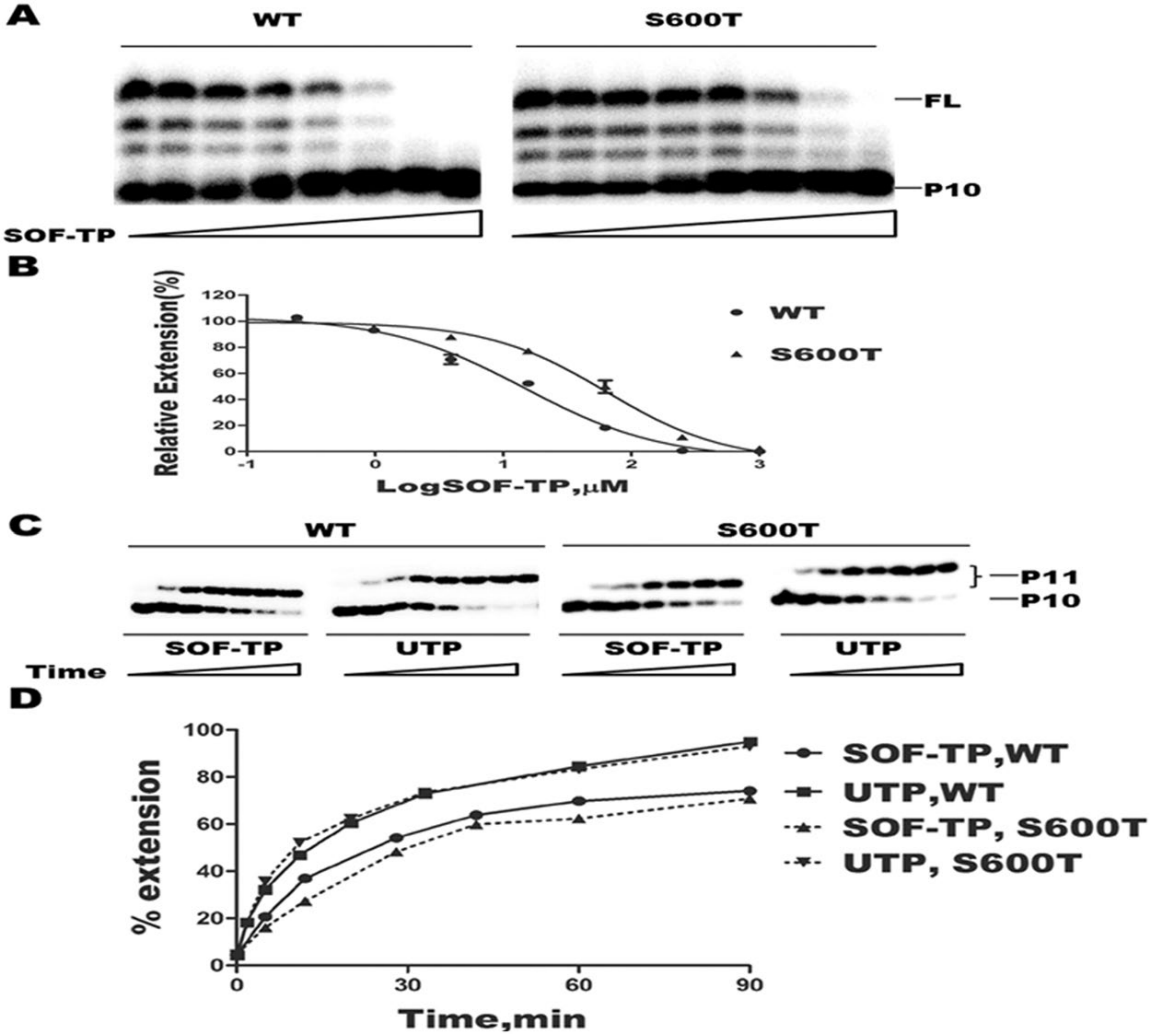
Inhibition of DENV NS5 RdRp activity by SOF-TP and time course of incorporation of SOF-TP or UTP in primer extension assays by DENV WT NS5 and the S600T mutant. (**A**) Inhibitory effect was determined by *in vitro* primer extension assay using purified NS5 protein**s** and P10/T14 RNA duplex template in RdRp assay buffer in the presence of 0.5 µM each of NTPs and variable concentrations of SOF-TP. Reaction products were analyzed by denaturing polyacrylamide gel electrophoresis (6% acrylamide, 7 M Urea). P10 and FL indicate the positions of the 5′-end radiolabelled P10 primer and full-length extension products of the primer extension reactions. (**B**) Dose-response curves showing inhibition by SOF-TP of the activity of DENV NS5 WT and the S600T mutant. The IC50 (50% inhibitory concentration) value was defined as the concentration of SOF-TP at which the full-length (FL) products were decreased by 50% relative to the control reaction performed in the absence of SOF-TP, and was calculated by nonlinear regression analysis: IC_50_WT = 14.7 ± 2.5 µM, IC_50_S600T = 63.8 ± 5.3 µM. The data represent the means ± standard deviation (SD) of 3 independent experiments. (**C**) The RNA T14 template and 5′-end radiolabeled RNA primer P10 duplex were used to assess the incorporation efficiency of UTP and SOF-TP on RNA synthesis by use of purified recombinant DENV NS5 WT and S600T mutant enzymes in time course primer extension assays over a period of 90 min. Sampling time points for UTP (min): 0.2, 2, 5, 10, 20, 30, 60, 90; SOF-TP (min): 0.2, 5, 15, 30, 45, 60, 90.P10 and P11 indicate the positions of the unextended and extended primers, respectively. (**D**) Quantitative analysis of UTP or SOF-TP incorporation in a time course primer extension assay. Incorporation efficiencies were compared based on the percentages of extended primer.

We next used the *in vitro* primer extension assay to determine the inhibitory effects of SOF-TP on DENV NS5 polymerase activity in the presence of all four natural NTPs. We first incubated the RNA duplex P10/T14 with purified DENV NS5 WT or S600T mutant enzymes in the presence of variable concentrations of SOF-TP in RdRp assay buffer at 30 °C for 10 min. Reactions were initiated by adding 0.5 µM each of NTPs and were stopped after 50 min. The S600T mutant was chosen for this study based on the fact that S600T is highly conserved in the viral RdRp B motif and was previously shown to confer resistance to nucleoside analogue inhibitors^16^. Reaction products were mixed with 2 volumes of formamide loading buffer, separated by denaturing polyacrylamide gel electrophoresis (6% acrylamide, 7 M urea) and analyzed by phosphorimaging (Fig. 4A). The 50% inhibitory concentration (IC_50_), which was defined as the concentration of SOF-TP at which the full-length (FL) products were decreased by 50% relative to the control reaction in the absence of SOF-TP, was determined by nonlinear regression analysis using GraphPad Prism5.0 software (GraphPad Software, San Diego, CA). We showed that SOF-TP was active against DENV NS5 WT polymerase activity with an IC_50_ value of 14.7 ± 2.5 µM. In contrast, the S600T substitution conferred a 4.3-fold resistance to SOF-TP with an IC_50_ value of 63.8 ± 5.3 µM (Fig. 4B). This result indicates that the S600T substitution in DENV NS5, which corresponds respectively to the S604T and S282T substitutions in the B motifs of the ZIKV NS5 and HCV NS5B, that confer resistance to nucleotide inhibitors^27, 37^, also confers resistance to SOF-TP. These data demonstrate that the B Motif of viral RdRp enzymes play a significant role in the ability of 2′-modified nucleotide analogues to inhibit viral RdRp activity.

### The DENV NS5 S600T mutant decreases the incorporation efficiency of SOF-TP but not of UTP

To understand the mechanisms involved in the resistance conferred by S600T against SOF-TP, we performed time-course single-nucleotide incorporation reactions in the presence of saturating concentration of UTP or SOF-TP in a primer extension assay using P10/T14 RNA duplex using both purified DENV WT NS5 and the S600T mutant enzyme. We first incubated the RNA duplex P10/T14 with purified DENV NS5 WT or S600T mutant enzymes in RdRp assay buffer at 30 °C for 10 min. Reactions were initiated by adding 5 µM of UTP or 1 mM SOF-TP. Samples were removed at variable time points over a period of 90 min and mixed with 2 volumes of formamide loading buffer, separated by denaturing polyacrylamide gel electrophoresis (20% acrylamide, 7 M urea) and analyzed by phosphorimaging (Fig. 4C). The percentage of extended primer P11 was plotted over time (Fig. 4D). The data shown in Fig. 4D indicate that the S600T mutant incorporated the natural UTP with the same efficiency as did WT enzyme. In contrast,, the S600T mutant enzyme incorporated SOF-TP less efficiently than did WT as demonstrated by the lower percentage of P11 that was formed over the 90 min of reaction. This result shows that the presence of S600T results in decreased incorporation efficiency of SOF-TP.

### The incorporated 3′-terminal nucleotide SOF analogue on nascent RNA cannot be excised by DENV NS5 in the presence of physiological concentrations of ATP

**Figure 5:**
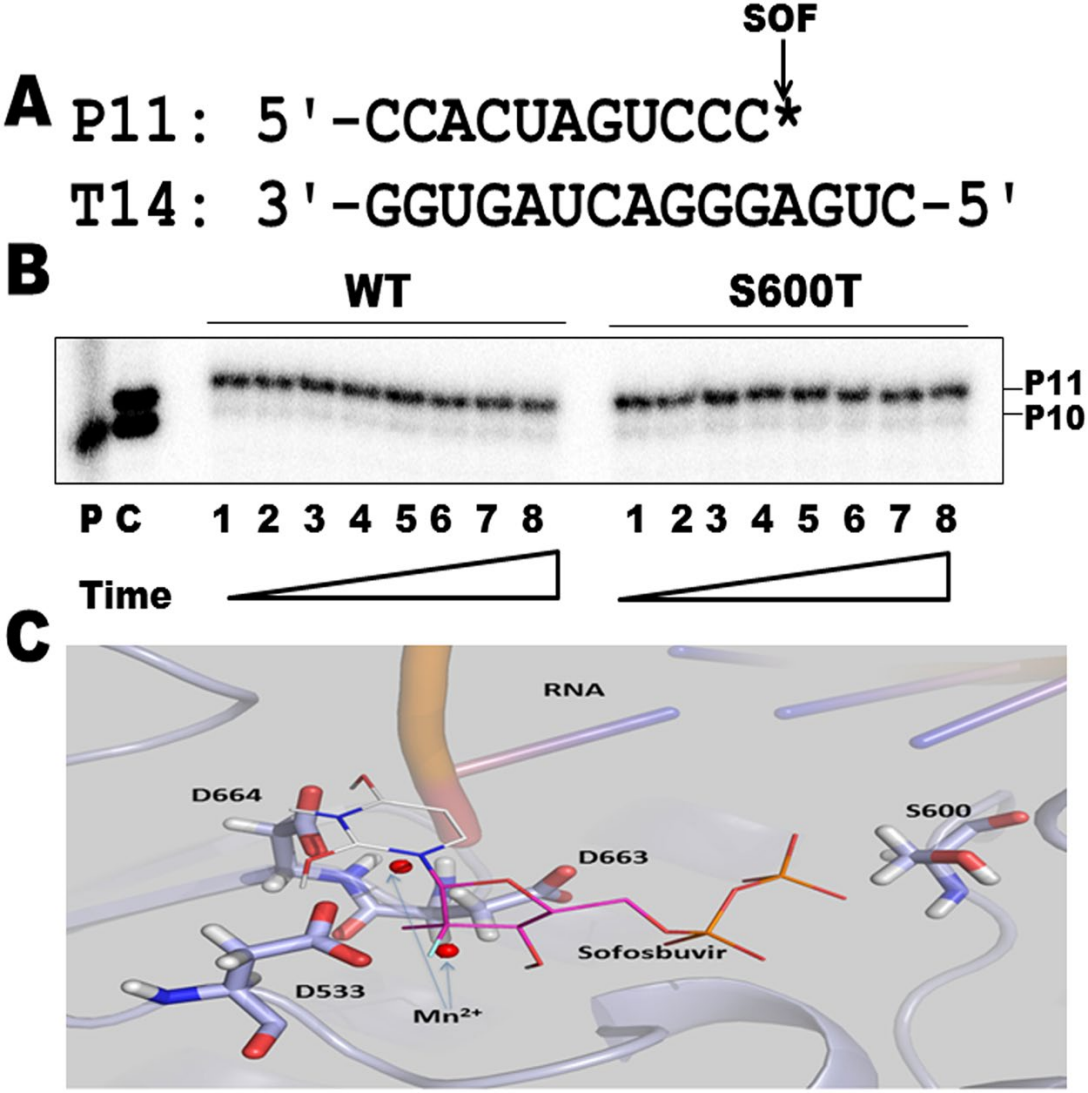
ATP-dependent excision of 3′ SOF-terminated primer and docking of SOF-TP into the active site of DENV polymerase in a homology model. (**A**) Sequences of the SOF-terminated RNA primer P11 RNA and the RNA template T14 used in the ATP-dependent excision assay. The position of incorporated SOF is indicated by “*”. (**B**) Time course of the excision reaction. The SOF-terminated primer P11/ RNA template T14was incubated with DENV NS5 WT or S600T mutant in RdRp assay buffer in the presence of 3.2 mM ATP. An aliquot of reaction mixture was removed at various time points. Lanes 1 to 8 represent time points at 15, 30, 45, 60, 75, 90, 105, 120 min. P: 5′-end radiolabeled RNA primer P10. (**C)** partially extended RNA primer P10 by incorporation of SOF-TP at the +1 position as size markers. Samples were analyzed using denaturing PAGE (20% acrylamide, 7 M Urea) and phosphorimaging. Positions of primers P10 and P11 are indicated on the right.(C) Catalytic residues D533, D663, and D664 are in close proximity to coordinate both Mn^2+^ cations (red circles). SOF-TP is shown in lines. The catalytic triad residues D533, D663, and D664 are shown in sticks. Mn^2+^ cation binding was mimicked by direct structure overlay from the HCV NS5B crystal structure (PDB ID 4WTG). A short double stranded RNA was retrieved from the PDB structure 4WTG and overlaid into the homology model structure. Structure visualization was performed using PyMOL.

Excision of incorporated nucleotide analogue chain-terminators as the result of pyrophosphorolysis may diminish their inhibitory effects on viral RNA-dependent RNA polymerases^38–40^. ATP-mediated excision has been demonstrated for HCV NS5B^40^, but was not detected for the NS5B of bovine viral diarrhea virus (BVDV)^38^, a member of the same Flaviviridae family to which HCV and DENV belong. To determine whether incorporated SOF can be excised by DENV NS5, we incubated the chain-terminated RNA duplex P11/T14 (Fig. 5A) with purified DENV NS5 WT or S600T mutant enzymes in the presence of 3.2 mM ATP and monitored the reactions over two hours. No excision was detected (Fig. 5B). Considering that the excision reaction may be impacted by the length of the RNA duplex, we repeated the excision experiments using an extended RNA P21/T24 duplex and obtained similar results, ie no excision was observed (data not shown). In addition, the same results were obtained in the presence of physiological concentration (150 µM) of pyrophosphate (PPi) (data not shown). It may be likely that minor differences in the structure and function of these viral RNA polymerases from different members of the Flaviviridae family are responsible for these differences.

### Molecular docking

Based on our homology model (Fig. 5C), SOF-TP fits well into the active site of DENV NS5 and the amino acid residues relevant to the SOF-TP interaction are critical for natural ribonucleotide incorporation. The model supports the biochemical data above that showed that SOF-TP can be recognized as an effective substrate and incorporated into nascent RNA chain by DENV polymerase. The catalytic residues D533, D663, and D664 coordinate both Mn^2+^ ions, inferring that they can prime DENV polymerase for initiation and elongation of RNA synthesis. Residue S600 is within a short interaction distance from SOF-TP.

## Discussion

Here, we have shown that SOF can inhibit Dengue virus replication in cell culture. In addition, we have characterized SOF-TP using purified Dengue virus NS5 in primer extension assays.

First, we have demonstrated that this uridine analogue is an active substrate for Dengue virus polymerase and can be incorporated into nascent RNA chains to cause chain termination. However, SOF-TP is a less efficient nucleotide substrate relative to the natural UTP in a single nucleotide incorporation assay. Second, we have shown that SOF-TP inhibits DENV NS5 polymerase activity with an IC_50_ value of 14.7 µM in the presence of competing 0.5 µM UTP, which is a little higher than its K_1/2_ value. We have also demonstrated for the first time that a S600T substitution within DENV NS5 confers 4.3 fold-resistance against SOF-TP and that the mechanism of this resistance is a decrease in the incorporation efficiency of SOF-TP. Finally, we have uniquely shown that incorporated SOF-TP cannot be excised by either DENV NS5 WT enzyme or by the S600T mutant. Importantly, our study has utilized both cell-based and biochemical approaches to show that although SOF was not efficiently incorporated into nascent RNA chains it was still able to impair virus replication. This is important since SOF is a clinically approved drug with a good safety profile. As there is no approved direct-acting antiviral agent to treat severe Dengue virus infections, SOF may be a reasonable therapeutic candidate for severe life-threatening cases of Dengue. Besides, SOF may act as a good starting point for medicinal chemistry efforts for the development of more specific and potent therapeutic anti-dengue nucleotide inhibitors.

It should be noted that unlike HCV infection which is chronic where treatment lasts over weeks and thus quick kinetics of drug concentration and a high potency against the virus are not necessarily required for success, severe dengue infection is an acute disease requiring rapid acting and highly effective antivirals. It is possible that the relative lower potency of SOF against Dengue virus infection compared to HCV infection may limit clinical application of SOF on severe cases of Dengue. Nonetheless, no untoward effects were observed in test subjects administered by over dosage of sofosbuvir at a single dose of 1200 mg, three times the FDA-recommended dosage (https://www.accessdata.fda.gov/drugsatfda_docs/label/2015/204671s002lbl.pdf, accessed on May 18, 2017). Administering higher but safe dosage of SOF in certain severe cases of Dengue may be a potential therapeutic option for achieving the goal of rapid virologic suppression and decreasing mortality.

Single nucleotide incorporation assays have shown that the incorporation of SOF-TP by both Zika virus NS5 and DENV NS5 is very inefficient^35, 36^. However, both cell-based assays and animal studies have demonstrated that SOF can inhibit Zika virus replication^26–28^. This discrepancy between single-nucleotide incorporation enzymatic assays and cell culture and animal model data suggest that the high discrimination values of certain nucleotide analogues in single-nucleotide incorporation assays may not necessarily preclude antiviral activity in cells. In order to clarify this issue in the case of DENV, we showed in single-nucleotide incorporation assays with DENV NS5 that SOF-TP is less efficiently incorporated by DENV NS5 than UTP, in agreement with previous reports^35, 36^. However, our cell based assays have shown that SOF can efficiently inhibit Dengue virus replication. In addition, our primer extension assays showed that SOF-TP can inhibit DENV NS5 polymerase activity with an IC_50_ of 14.7 µM in the presence of 0.5 µM competing UTP, close to its K_1/2_ value 0.4 μM. One explanation for any discrepancy may be that SOF-TP can be incorporated at multiple sites during the intracellular replication of the ~11 kb viral genome RNA to cause RNA chain termination, resulting in defective viral RNA genomes. In addition, although chain-termination is the major mechanism for the activity of 2′-modified ribonucleotide analogues such as SOF^22, 24^, the incorporation of 2′-modified ribonucleotide analogues may also have the potential to have a mutagenic effect, such that mutagenesis occurs in a manner similar to that observed for the broad spectrum antiviral ribonucleotide analogue Ribavirin^41, 42^. The Zika virus genome under Sofosbuvir pressure showed higher frequencies of overall transition mutations, mainly A-to-G changes, compared to control sequences in cell cultures^28^. It is possible that the DENV genome may also exhibit A to G hypermutagenesis under SOF pressure. If so, the combined effects of SOF as both a direct-acting viral polymerase inhibitor and viral RNA mutagen may enhance its anti-DENV activity and that this could be underestimated in a single-nucleotide incorporation assay. In addition, the poor excision capability of incorporated SOF-TP by both WT and the S600T mutated DENV NS5 enzymes suggest that incorporation may lead to an irreversible halt of viral RNA synthesis. The lower incorporation efficiency of SOF-TP over UTP by Dengue virus NS5, which can limit the effectiveness of the inhibitor, may be not critical as incorporation of a single SOF-TP at any site acts as a fairly irreversible chain terminator. The lower incorporation efficiency of SOF-TP may also be overcome by potential local high concentration of SOF-TP at the site of Dengue virus replication.

Evidence showing that SOF provides protection in a lethal mouse model as demonstrated for other class of anti-dengue small molecule compounds^43^ is important for advancing this compound into clinical studies for treatment of severe DENV infections. A limitation of the current study is that we were not able to test the antiviral activity and toxicity of SOF in a DENV animal model; this will be carried out in future studies. SOF, as an approved drug for clinical treatment of chronic HCV infections, its safety has been well demonstrated in clinical trials and clinical applications^44, 45^.

Ongoing work is now assessing whether SOF possesses inhibitory effects against the polymerases of other flaviviruses including the clinically significant YFV and JEV. Further research will also assess the binding modes of SOF-TP to the active sites of wild-type and mutated DENV NS5 and HCV mutant enzymes, so that more effective DENV NS5 inhibitors can be designed based on structure-function relationships.

## Methods

### Compounds and nucleic acids

All DNA and RNA oligonucleotides used in this study were purchased from Integrated DNA Technologies (IDT DNA, Coralville, IA). For 5′-end labelling of oligonucleotides with [γ32 P]ATP, the Ambion KinaseMax kit was used followed by purification through Ambion NucAway spin columns, according to protocols provided by the supplier (Thermo Fisher Scientific Inc, Mississauga, ON).

Sofosbuvir (SOF) and its active triphosphate form (SOF-TP) were kindly provided by Gilead Sciences Inc. Mycophenolic acid (MPA) was obtained from Sigma-Aldrich (Markham, ON).

### Determination of antiviral activity and cytotoxicity by cytopathic effect protection assay

The DENV2 NGC viral strain and Huh-7 (human hepatocellular carcinoma) cells were used in our cell-based antiviral assays. The viral stock was prepared by inoculation of C6/36 cells (*Aedes albopictus* clone C6/36 cells; ATCC CRL166) as described^29, 46^. Cytopathic effect protection assays for evaluation of antiviral activity against DENV were performed using the Promega Viral ToxGlo Assay (Thermo Fisher Scientific Inc, Mississauga, ON) as described previously with minor modifications^27–29^. Briefly, Huh-7 cells were plated into 96 well plates at 10,000 cells/well in DMEM containing 10% FBS and 1% penicillin/streptomycin. Cells were incubated at 37 °C overnight and infected with DENV for 90 min as described previously^29^. After absorption, the inoculum was removed by aspiration, the monolayers were washed twice with DMEM containing 1% penicillin/streptomycin, and fresh DMEM medium containing 2.5% FBS, and 1% penicillin/streptomycin with variable concentrations of test compounds was added. Cell cultures were maintained at 37 °C in a 5% CO_2_ incubator for 2 days. At 24 hours post infection, half of the medium was removed and replaced by fresh medium containing the same concentrations of test compounds. The Viral ToxGlo Reagent was prepared fresh and 100 µl reagent were added to each well. Plates were incubated for at least 10 minutes and then quantitated by a 1450 MicroBeta TriLux Microplate Scintillation and Luminescence Counter (Wallac-PerkinElmer Inc., Wellesley, MA). For cytotoxicity assays, test compounds were processed and added to uninfected cells in the same way. CC_50_ (50% cytotoxic concentration) values were determined using the Viral ToxGlo Assay. Dose-response data were analyzed by nonlinear regression using GraphPad Prism5.0 software (GraphPad Software, San Diego, CA).

### Determination of anti-dengue virus activity of SOF by virus yield reduction (VYR)_assay

Huh7 cells were seeded as above into 96-well plates the day before infection. Cells were infected with a low dose of Dengue virus (multiplicity of infection = 0.01) and treated with variable concentrations of SOF as described above in CPE protection assay. Viral titers in the cell culture supernatant quantified as the 50% tissue culture infective dose (TCID_50_) were determined by the Promega Viral ToxGlo Assay (Fisher Scientific, Ottawa, ON, Canada) using Huh7 cells as instructed by the manufacturer. Infected cells without SOF treatment served as control. Dose-response data were analyzed using GraphPad Prism5.0 software to calculate EC_50_ and 90% effective concentration (EC_90_) (GraphPad Software, San Diego, CA) as instructed by the manufacturer.

### Viral RNA extraction and quantitative real-time reverse transcription PCR

The dose-dependent impact of SOF on DENV viral RNA production was measured by quantitative real-time reverse transcription PCR (qRT-PCR). Viral RNA was purified from cell culture supernatants using the QIAamp® Viral RNA Mini Kit according to the manufacturer’s instructions (Qiagen, Mississauga, ON). The qRT-PCR was performed with Invitrogen SuperScript III Platinum One-Step qRT-PCR Kit according to manufacturer’s instructions (Thermo Fisher Scientific Inc, Mississauga, ON). Primers and probe sequences were derived from the DENV NS5 polymerase gene:

DENV2qPCRF: 5′- CACACATGAGATGTACTGGGTATC-3′,

DENV2qPCRR: 5′-GGCTCGTAAGTGGCTTTCTT-3′,

DENV2Probe:5′-/56-FAM/AATGCCTCC/ZEN/GGGAACATAGTGTCA/3IABkFQ/-3′. All amplifications were performed in a Rotor-Gene 6000 real-time thermo cycler (Corbett Life Science, Concorde, NSW, Australia) with the following cycles: reverse transcription at 50 °C for 15 min, initial denaturation at 95 °C for 120 s, followed by 50 cycles of 95 °C for 15 s and 60 °C for 30 s. Dose-response data were analyzed by nonlinear regression using GraphPad Prism5.0 software (GraphPad Software, San Diego, CA).

### Construction of DENV NS5 bacterial expression plasmids

The gene encoding full-length NS5 of DENV2 was amplified by PCR from plasmid pBAC-DENV-FL DNA (kindly provided by Drs. Jose A. Usme-Ciro and Juan C. Gallego-Gomez, Universidad de Antioquia, Colombia) which contains the full length cDNA of the DENV2 NGC strain^47^, and was cloned into the Novagen pET21a vector (EMD Millipore, Etobicoke, ON) to produce pET21aDENV_2_NS_5_FLcHis_6_ for expression and purification of recombinant DENV NS5 containing a carboxyl-terminus hexahistidine tag in *E. coli*. The amino acid substitution S600T in motif B of DENV RdRp was introduced into Dengue virus NS5 using a Quick-change mutagenesis kit (Agilent Technologies Canada Inc., Mississauga, ON).

### Expression and purification of recombinant Dengue virus NS5

Novagen BL21 (DE3) competent cells (EMD Millipore, Etobicoke, ON, Canada) were transformed with the pET21aDENV2NS5FL plasmid for expression and purification of the DENV NS5 with a C-terminal LEHHHHHH tag using minor modifications of published protocols^29, 33, 48^. Briefly, the bacterial cells were cultured in LB medium supplemented with carbenicillin (100 µg/ml) at 37 °C until the OD600 reached 0.6–0.8. After induction of expression with 0.4 mM isopropyl β-D-1-thiogalactopyranoside (IPTG) in the presence of 2% ethanol, the cells were grown overnight at 15 °C with a shaking speed of 250 rpm. Cells were harvested by centrifugation, resuspended in lysis buffer containing 50 mM Tris-HCL pH7.5, 500 mM NaCl, 5 mM 2-mercaptoethanol, 1% Igepal-CA630, 20% glycerol, 1 mM phenylmethylsulfonylfluoride (PMSF), 15 mM imidazole and 1 x complete EDTA-free protease inhibitor cocktail (Roche, Mississauga, ON). Cells were lysed by sonication and clarified by centrifugation. The clarified supernatant was loaded onto a Novagen nickel-nitrilotriacetic (Ni-NTA) column (EMD Millipore, Etobicoke, ON) equilibrated with lysis buffer. The Ni-NTA column was washed with lysis buffer containing 30 mM imidazole and eluted with lysis buffer containing a gradient of imidazole from 60 mM to 300 mM. Fractions containing NS5 were combined and then applied onto a Superdex 200 gel filtration column (GE Healthcare Life Sciences, Brampton, ON). After the gel filtration step, protein fractions containing NS5 were combined, diluted in a buffer containing 50 mM Tris, pH 7.0, 300 mM NaCl, 5 mM TCEP (tris(2-carboxyl)phosphine), 20% glycerol and concentrated by Amicon Ultra-15 Centrifugal Filter Units (MWCO 30,000, EMD Millipore, Etobicoke, ON, Canada). Protein concentrations were measured by a Bradford protein assay kit (Bio-Rad Laboratories, Saint-Laurent, QC, Canada) using bovine serum albumin (BSA) as a standard.

### Primer extension assay for nucleotide analogue incorporation and chain termination

Primer extension reactions were performed using RNA primer P10: 5′-CACUAGUCCC-3′ and RNA template T14: 5′- CUGAGGGACUAGUG-3′. P10 was annealed to T14 (molar ration of P10:T14 = 1:1.5) in a buffer containing 50 mM Tris pH 7.0 and 50 mM NaCL by heating at 95 °C for 5 min and slowly cooling to room temperature. Standard reaction mixtures consisted of 25 nM RNA primer/template duplex and 50 nM NS5 protein in RdRp assay buffer containing 40 mM Tris-HCL, pH 7.0, 10 mM NaCL, 5 mM KCL, 1 mM MgCl_2_ and 0.5 mM MnCL_2_, 0.001% Triton X-100, and 10 µM cysteine. Reactions were initiated by the addition of nucleotide (nt)/nt-analogue, 5 µM of UTP or 200 µM SOF-TP in the absence or presence of 5 µM next incoming nucleotide CTP. Reactions were carried out for 10 min for UTP or 50 min for SOF-TP incorporation at 30 °C, which is the optimal temperature for maximal enzyme activity of purified NS5 protein. For comparison of incorporation of SOF-TP or UTP in a time-course primer extension assays by DENV WT NS5 and the S600T mutant, the reactions were initiated by adding 5 µM UTP or 1 mM SOF-TP and were allowed to proceed for 90 min. Reactions were stopped by adding 2 volumes of formamide loading buffer (90% formamide, 10 mM EDTA, and 0.1% each of xylene cyanol and bromophenol blue). Reaction products were denatured by heating at 95 °C for 5 min and analyzed using denaturing polyacrylamide gel electrophoresis (PAGE, 7 M Urea-20% or 6% acrylamide as indicated in the figure legends). The gels were dried at 80 °C before phosphorimaging. Band intensities were quantified with ImageQuant software (GE Healthcare, Brampton, ON), and data analyses were performed using GraphPad Prism5.0 software (GraphPad Software, San Diego, CA).

### ATP-dependent excision assay

Chain-termination and excision reactions were performed as described with modifications^39, 40, 49^. Briefly, 5′-end radiolabelled RNA primer P10 was annealed with excess RNA template T14 (molar ratio 1:3) and used for chain termination reactions. Reaction mixtures consisted of 1 µM P10/T14 RNA duplex (P10 concentration), 1.5 µM DENV NS5 in RdRp assay buffer containing 40 mM Tris-HCL, pH 7.0, 10 mM NaCL, 5 mM KCL, 1 mM MgCl_2_ and 0.5 mM MnCL_2_, 0.001% Triton X-100, and 10 µM cysteine. Chain termination reactions were initiated by adding 250 µM of SOF-TP and incubated at 30 °C for 120 min. Reaction products were extracted with phenol/chloroform, precipitated by ethanol and resuspended in a buffer containing 50 mM Tris pH7.0 and 50 mM NaCL. Complete chain termination was verified by standard primer extension assay in the presence of 20 µM each of NTPs, such that no extension was observed after the terminated primer (data not shown).

For the ATP-dependent excision reaction, chain-terminated P11/T14 was incubated with 1.5 µM wild-type (WT) or mutant DENV NS5 enzymes in RdRp assay buffer at 30 °C. The reaction was initiated by adding 3.2 mM of ATP and aliquots of reaction mixture were removed at variable time points over a period of 120 min, mixed with 2 volumes of formamide loading buffer, analyzed by denaturing polyacrylamide gel electrophoresis (20% acrylamide, 7 M urea) and phosphorimaging.

### Molecular modeling

A homology model of dengue RdRp was generated using the I-TASSER protein structure prediction server^50, 51^. Previously published structures were obtained through the Research Collaboratory for Structural Bioinformatics (RCSB) Protein Data Bank (http://www.rcsb.org/pdb/). Molecular modeling was performed using the published lead template of DENV NS5 polymerase (PDB: #5DTO)^10^. The published structures of HCV NS5B in complex with Mn^2+^ and primer template (PDB ID: 4WTA; 4WTG) were also used for the generation of the dengue polymerase homology model^23^. The structure of SOF-TP was retrieved from published crystal structure data (PDB ID: 4WTG) and used as a ligand^23^. Minimization of ligand-docked structures was facilitated by the use of a UCSF Chimera (http://www.cgl.ucsf.edu/chimera/)^52^, which allowed input of DENV polymerase and SOF-TP for DOCK calculations, which predict the orientation of the ligand. Docking calculations were performed using AutoDock Vina^53^, within the virtual screening tool PyRx 0.8 (http://pyrx.scripps.edu). A grid box centered on catalytic residues D533 and D663 was used to include all possible docking sites in DENV polymerase. Docking results were processed to identify intermolecular interactions using the structural visualization and image processing software PyMOL (http://www.pymol.org). Model quality was assessed based on root mean square deviation (RMSD) of the global homology structure using the RCSB PDB Protein Comparison Tool. The putative positions of Mn^2+^ and RNA were superimposed from structures 4WTG and 4WTA respectively. The homology model was verified by Ramachandran plot analysis to have greater than 98.0% of residues in allowed and favourable orientation^54^.

## Electronic supplementary material

Supplementary Info File #1
