# Supplementary material for: Evaluation of Sofosbuvir (β-D-2′-deoxy-2′-α-fluoro-2′-β-C-methyluridine) as an inhibitor of Dengue virus replication#
Source: Sci Rep. 2017 Jul 24;7:6345. doi: 10.1038/s41598-017-06612-2 (PMC5524696; doi:10.1038/s41598-017-06612-2)

**Evaluation of Sofosbuvir (β-D-2’-deoxy-2’-α-fluoro-2’-β-C-methyluridine) as an inhibitor of Dengue virus replication^#^**

Hong-Tao Xu^1*^, Susan P. Colby-Germinario^1^, Said A. Hassounah^1^, Clare Fogarty^1^, Nathan Osman^1^, Navaneethan Palanisamy^1,3,4^, Yingshan Han^1^, Maureen Oliveira^1^, Yudong Quan^1^, and Mark A. Wainberg^1,2,3^

Supplementary Fig. 1. Parallel comparisons of incorporation of natural UTP (5 µM for 10 min) or nucleotide analogue SOF-TP (200 µM for 50 min ) into RNA and chain-termination by purified recombinant DENV NS5 were performed at 30 ^o^C in a primer extension assay.


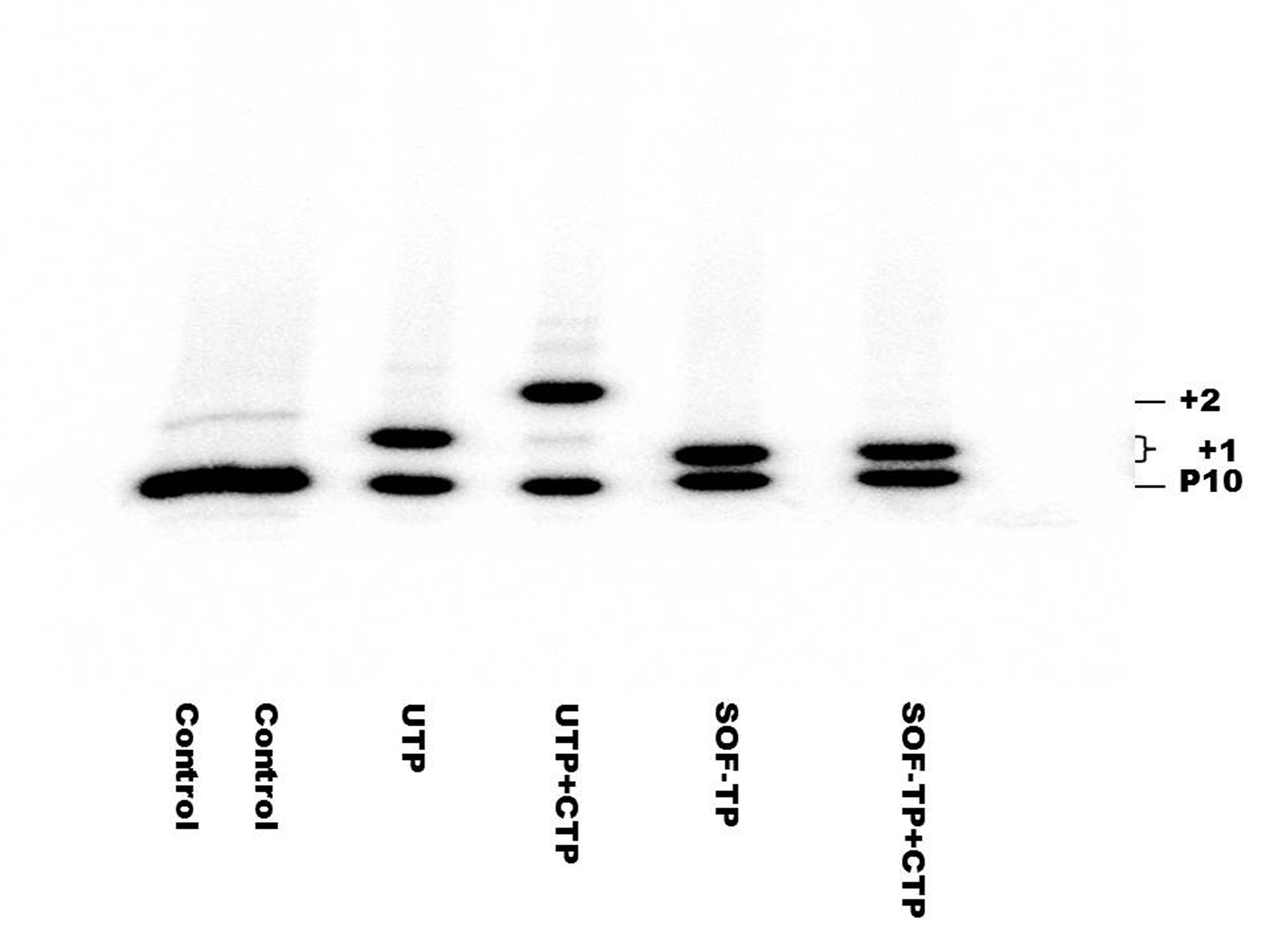


Supplementary Fig. 2. Single nucleotide incorporation reactions were performed at 30 ^o^C in a standard primer extension assay using RNA duplex P10/P11 and DENV NS5 in the presence of variable concentrations of the nucleotide analogue SOF-TP.


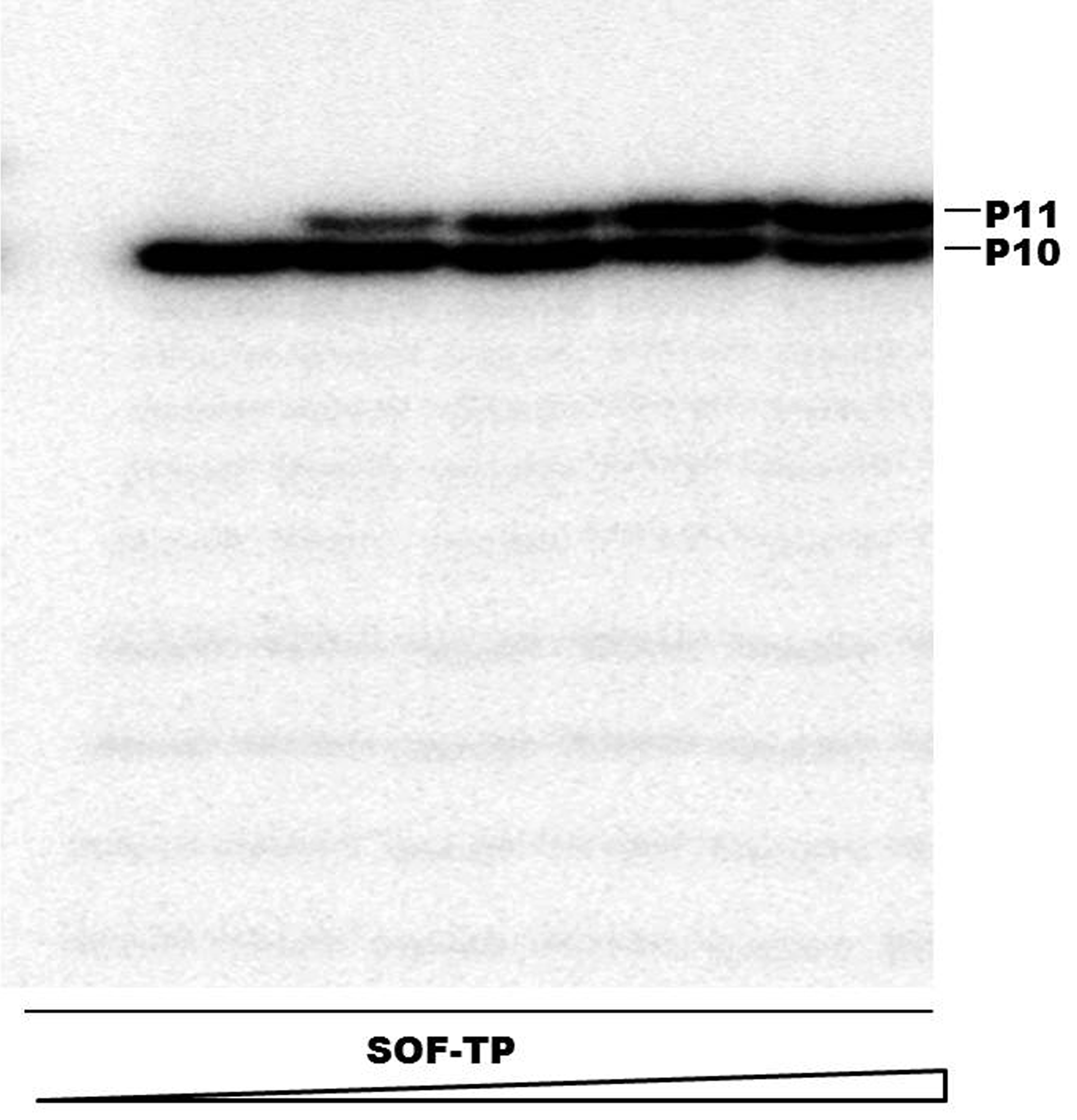


Supplementary Fig. 3. Single nucleotide incorporation reactions were performed at 30 ^o^C in a standard primer extension assay using RNA duplex P10/P11 and DENV NS5 in the presence of variable concentrations of natural nucleotide UTP.


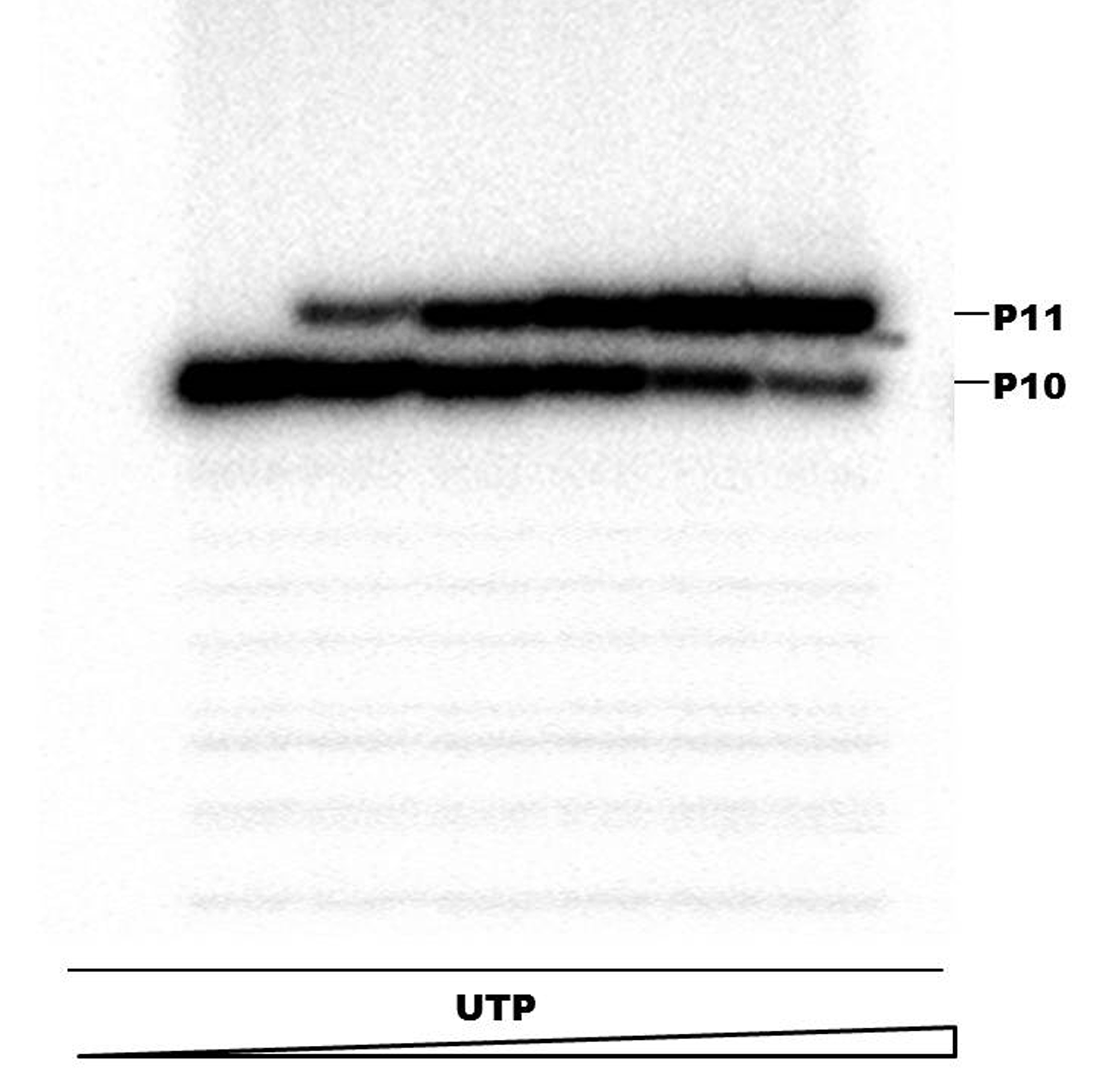


Supplementary Fig. 4. Time course of ATP-dependent excision of 3’ SOF-terminated primer.


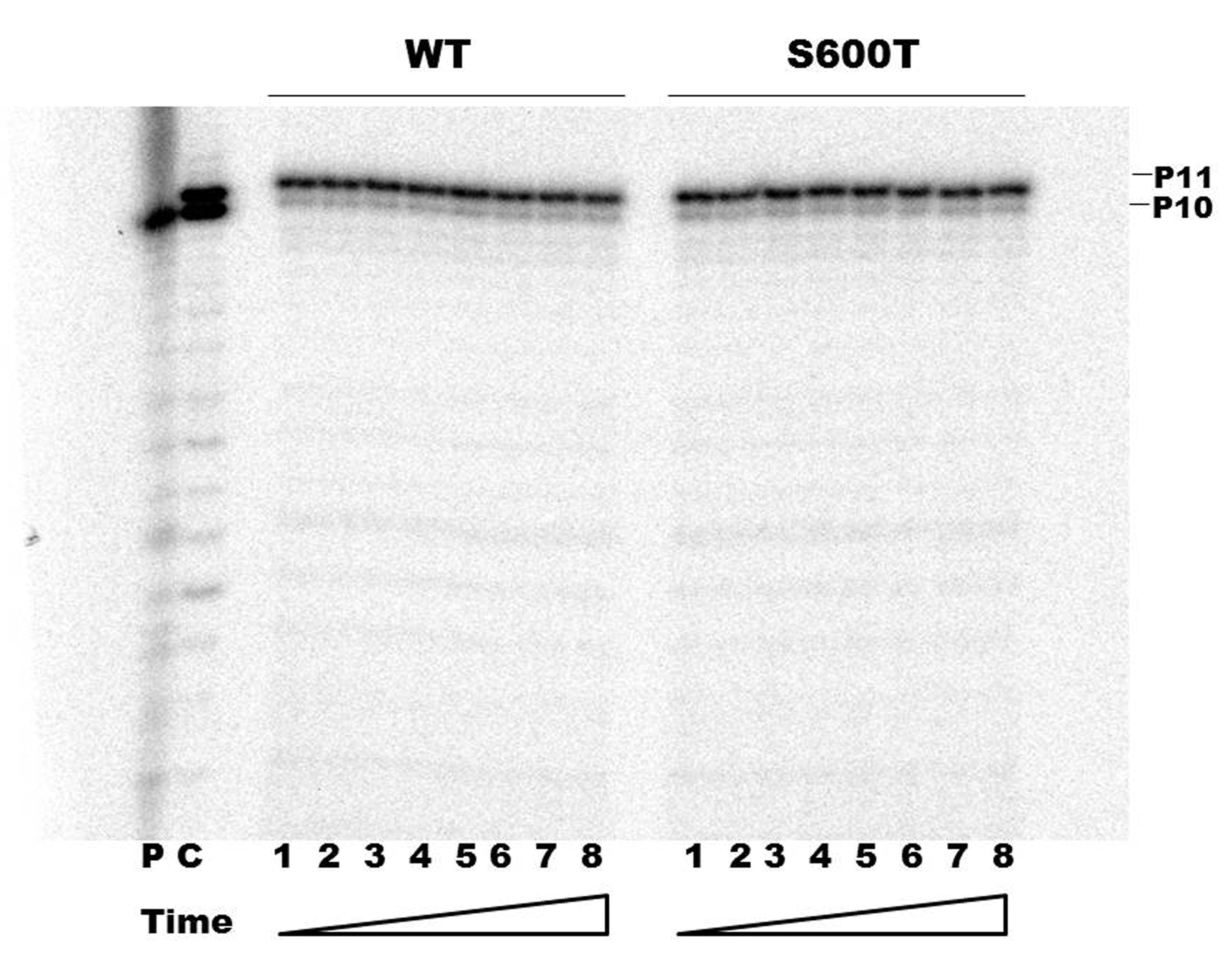

Supplement: Supplementary file 1 — Supplementary Info File #1 [file 41598_2017_6612_MOESM1_ESM.docx]
